# Impact of the intensity of infection in birds on *Plasmodium* development within *Culex pipiens* mosquitoes

**DOI:** 10.1186/s13071-024-06652-4

**Published:** 2025-02-14

**Authors:** Julie Isaïa, Molly Baur, Jérôme Wassef, Sarah Monod, Olivier Glaizot, Philippe Christe, Romain Pigeault

**Affiliations:** 1 Department of Ecology and Evolution, University of Lausanne, Lausanne, Switzerland; 2 Department of Zoology, State Museum of Natural Sciences, Lausanne, Switzerland; 3 Ecologie & Biologie Des Interactions (UMR 7267), EBI, Université de Poitiers, Poitiers, France

**Keywords:** Aggregated distribution, Avian malaria, Extrinsic incubation period, EIP, Transmission, Overdispersion

## Abstract

**Background:**

In vector-borne diseases, invertebrate hosts are exposed to highly variable quantities of parasites during their blood meal. This heterogeneity may partly explain the overdispersed distribution of parasites within the vector population and the variability in the extrinsic incubation period (EIP) of the parasite. Indeed, the quantity of parasites ingested is often considered as a good predictor of the quantity of parasites that will develop within the vectors, as well as the speed at which they will develop (i.e. EIP). However, density-dependent processes can influence the relationship between parasite burden in the vertebrate host and in vectors, making this relationship unclear at times.

**Methods:**

Here, we used an avian malaria system to investigate whether the proportion of red blood cells infected by sexual and/or asexual stages of *Plasmodium relictum* influences the intensity of infection and the EIP within vectors. For this purpose, we experimentally infected 12 birds in order to generate a range of infection intensity. More than 1000 mosquitoes took a blood meal on these hosts, and the development of *Plasmodium* within the vectors was followed for more than 20 days.

**Results:**

Our study reveals a negative relationship between the intensity of infection in birds and the time until 10% of mosquitoes become infectious (EIP_10_). A period of only 4 days was sufficient to detect sporozoites in at least 10% of mosquitoes fed on the most infected hosts. However, the number of sporozoites did not vary significantly according to the vertebrate host intensity of infection, but was positively correlated to the oocyst burden (parasitic stage preceding the sporozoite stage).

**Conclusions:**

While the quantity of ingested parasites had no impact on oocyst and sporozoite burden in infectious mosquitoes, the EIP_10_ was affected. Studies have demonstrated that small changes in the EIP can have a significant effect on the number of mosquitoes living long enough to transmit parasites. Here, we observed a difference of 4–6 days in the detection of the first sporozoites, depending on the intensity of infection of the bitten vertebrate host. Considering that a gonotrophic cycle lasts 3–4 days, the shortened EIP may have significant effects on *Plasmodium* transmission.

**Graphical Abstract:**

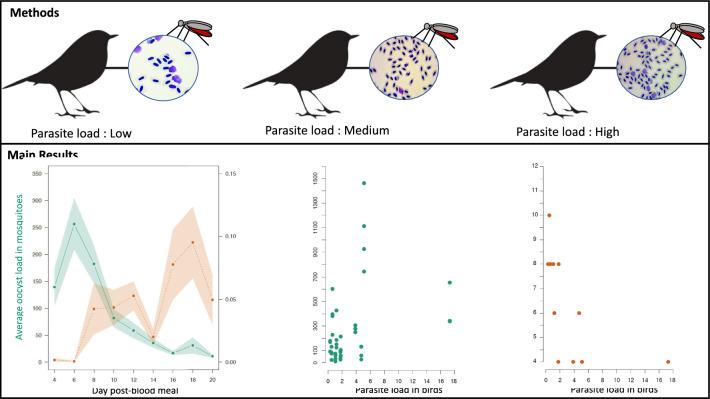

**Supplementary Information:**

The online version contains supplementary material available at 10.1186/s13071-024-06652-4.

## Background

The rate at which a population grows or declines is influenced not only by environmental conditions but also by the number of individuals already present in the population. This phenomenon, studied in population ecology, is known as a density-dependent process [1–4]. Density-dependent mechanisms are considered of central importance in stabilizing the population growth of both free-living [5] and parasitic organisms [6, 7]. Parasites are especially subject to density-dependent processes, as they often have complex life cycles involving bottlenecks upon transmission followed by exponential growth [2, 7–9]. Both positive and negative density-dependent processes are common in host–parasite associations, and both may influence the parasite transmission rate [10, 11] and resilience of infection and control interventions [12, 13]. Parasites are typically overdispersed within host populations (i.e. are aggregated, and the variance is considerably greater than the average [14, 15]), and the strength of density-dependent processes therefore differs greatly among hosts. Understanding the causes of parasite overdispersion is an important step towards assessing the impact of density-dependent processes on parasite transmission dynamics [16, 17].

Parasite overdispersion has been widely described in several vector-borne diseases [18–21]. However, most studies have focused on the causes and consequences of aggregate parasite distribution in vertebrate host populations, leaving aside what happens within invertebrate host populations (i.e. the vector). Yet, for many vector-borne diseases, key traits determining the transmission dynamics of the parasite (e.g. lifespan, feeding behaviour of vectors) may depend on the intensity of parasite infection in the vector [22–27]. Fluctuations in parasite density within vector populations may be due to the genetic background or age of the invertebrate hosts, which influence their susceptibility to parasite infection [18, 28, 29]. Environmental factors may also be involved, such as temperature, which affects the parasite’s ability to complete its life cycle within the invertebrate host [30, 31]. The number of parasites ingested by the vector during their infestation (usually a blood meal) can also generate infection heterogeneity (e.g. [32, 33]). Indeed, parasite numbers vary between vertebrate hosts and also spatio-temporally within individuals [34, 35]. Although a positive correlation is expected between the quantity of parasites present in the vertebrate host blood and the density of transmissible stages of the parasite found in vectors (after the incubation period), the relationship is not always obvious. This is particularly true for *Plasmodium* parasites, the aetiological agents of one of the most studied vector-borne diseases, malaria. The relationship between the proportion of red blood cells of the vertebrate host infected by gametocytes (i.e. the transmissible stage) and the parasite burden in the mosquito seems to be specific to *Plasmodium*–mosquito associations. For the human malaria parasite *Plasmodium falciparum* and both *Anopheles coluzzii* and *Anopheles gambiae* mosquitoes, the parasite burden within the mosquito midgut (i.e. the oocyst stage) increases with the density of gametocytes [36–39]. On the other hand, this relationship is either negative or unclear in the case of rodent and avian malaria, respectively [37, 40].

To date, the majority of the studies trying to elucidate the relationship between vertebrate host gametocytaemia (i.e. percentage of red blood cells infected by a mature gametocyte) and the parasite burden in mosquitoes only consider the oocyst stage, which is the simplest and least expensive measure of the mosquito infection. Even though oocyst burden seems to be positively correlated with the likelihood of transmitting sporozoites to a new vertebrate host [41], the extrinsic incubation period (EIP) of the parasite should be considered. EIP refers to the time required for the parasite to replicate and disseminate to the mosquito salivary glands after ingestion of the infected blood meal. It determines the time at which the vector will be infectious and able to transmit the parasite to a new vertebrate host: the shorter the EIP, the greater the opportunity for the mosquito to transmit the parasite [42].

The aim of this study was to assess the role of the vertebrate host parasitaemia (i.e. the percentage of red blood cells infected by either sexual or asexual stages of *Plasmodium*) and gametocytaemia on the within-vector parasite infection dynamics. We investigated whether the parasitaemia and gametocytaemia in the vertebrate host determine the density of oocysts in the mosquito midgut and/or the EIP of *Plasmodium*. Specifically, we studied the time for 10%, 50% and 90% of the infected mosquitoes to become infectious (denoted by EIP_10_, EIP_50_ and EIP_90_, respectively). We predicted that mosquitoes biting highly infected hosts would have higher oocyst burden and be infectious earlier than mosquitoes feeding on hosts with low parasitaemia and gametocytaemia. To test this prediction, we used a natural avian malaria system [40, 43], with domestic canaries (*Serinus canaria*) as the vertebrate host, a haemosporidian parasite recently isolated from the wild (*Plasmodium relictum*), and a natural vector of this parasite originally collected from a natural population of mosquitoes and reared under laboratory conditions thereafter (*Culex pipiens* [44]). Avian malaria is a relevant model for studying the ecology and evolution of host–parasite interactions, both in the field and in the laboratory. Indeed, in addition to the fact that avian malaria is widespread in birds and mosquitoes throughout the world, this biological system is amenable to laboratory experiments at every stage in the parasite’s life cycle (see [40, 43] for review).

## Methods

### Biological material

The experiment was carried out in May 2021 using a *P. relictum* (lineage SGS1) strain isolated from an infected house sparrow (*Passer domesticus)* captured in December 2020 on the campus of the University of Lausanne, Switzerland (46°31′25.607″N, 6°34′40.714″E). This strain was maintained through six regular passages across our stock canaries (*S. canaria*) using intraperitoneal (i.p.) injections until the beginning of the experiment [40].

The *Cx. pipiens* mosquito population used in this experiment was initiated from wild clutches collected in Lausanne in August 2017 and maintained in the insectary thereafter. Mosquitoes were reared using standard protocols [45]. On the day prior to a mosquito blood meal, around 1000 7–10-day-old females were randomly captured from different emergence cages and placed inside experimental cages (90 females per cage, 40 × 40 × 40 cm). During this time, females had access to water but were deprived of sugar solution, to maximize the biting rate.

### Experimental design

#### Infection of vertebrate hosts

Twelve domestic canaries (*S. canaria*) were infected with *P. relictum* (lineage SGS1) by i.p. from our stock canaries kept in the lab since January 2021, i.e. for 5 months prior to the experiment. One bird died on day 5 post-infection and was therefore removed from the analyses. The dynamics of infection were monitored for each bird every 2 days from day 5 to day 20 post-infection by measuring the parasitaemia and gametocytaemia by microscopic examination using Giemsa-stained blood smears. Blood samples (3–5 µl) were taken from the medial metatarsal vein. The number of red blood cells infected by sexual and/or asexual *Plasmodium* stages were counted per 3000–4000 erythrocytes in randomly chosen fields on the blood smears [46].

#### Mosquito exposure to *P. relictum*

On day 12 after vertebrate host infection, corresponding to the peak of infection for our parasite strain, birds were exposed individually to 90 uninfected mated female mosquitoes for 3 h (6–9 p.m.). At the end of the experiment, the number of blood-fed and unfed mosquitoes was counted, and only fully blood-fed females were kept individually in plastic tubes under standard laboratory conditions (25 °C, 70% relative humidity [RH]) with 10% glucose ad libitum. A total of 36 fully blood-fed mosquitoes per bird were used as samples for dissections (see Table S1 for details).

#### Mosquito dissections

Every 2 days starting from day 4 to day 20 post-blood meal, 4 ± 1 mosquitoes per bird were randomly sampled in order to monitor the dynamics of both oocyst formation and sporozoite production. Each mosquito was dissected to (i) count the number of oocysts in its midgut with the aid of a binocular microscope and (ii) quantify the sporozoites in its head/thorax using real-time quantitative polymerase chain reaction (qPCR; see molecular analyses below). The appearance of sporozoites in the head/thorax homogenate is strongly correlated with the appearance of sporozoites in the salivary glands [47]. The amount of haematin (a by-product of the decomposition of haemoglobin) deposited in the individual resting tubes was used to estimate the size of the blood meal. Quantification of the haematin was carried out by absorbance as described in previous papers [48]).

#### Molecular analyses

To study the presence/absence and the density of *Plasmodium* sporozoites in the mosquito head/thorax, qPCR was conducted. Beads were added to each head/thorax sample, after which the sample was flash-frozen in liquid nitrogen and then ground. Next, DNA was extracted using the Qiagen DNeasy Blood & Tissue Kit following the manufacturer’s instructions, except that the samples were incubated overnight. For each individual (i.e. mosquito), two qPCRs were carried out: one targeting the mitochondrial DNA (mtDNA) cytochrome b (cytb) gene of *Plasmodium* (primers L4050Plasmo 5′-GCTTTATGTATTGTATTTATAC-3′, H4121Plasmo 5′-GACTTAAAAGATTTGGATAG-3′, probe TexasRed-CYTB-BHQ2 5′-CCTTTAGGGTATGATACAGC-3′) and the other targeting the CQ11 gene of *Cx. pipiens* mosquitoes (primers 1725-F 5′-GCGGCCAAATATTGAGACTT-3′, 1726-R 5′-CGTCCTCAAACATCCAGACA-3′, probe FAM-CQ11-BHQ1 5′-GGAACATGTTGAGCTTCGGK-3′ [49]. All samples were run in triplicate (QuantStudio 6 and 7 Pro Real-Time PCR Systems), and samples with a cycle threshold (Ct) value higher than 35 for the parasite were considered uninfected. Relative quantification (RQ) values were calculated to assess the parasite prevalence and can be interpreted as the fold amount of the target gene (*Plasmodium* CYTB) with respect to the amount of the reference gene (*Cx. pipiens* CQ11), calculated as 2^−(C*t*CYTB*Plasmodium* −C*t*CQ11*Cx. pipiens*)^.

#### Statistical analyses

Analyses were carried out using R statistical software (v. 4.2.1). The relationship between the birds’ gametocytaemia and parasitaemia was studied using a linear regression. We verified the model’s assumptions by plotting residuals using the simulateResiduals function from the DHARMa [Residual Diagnostics for Hierarchical (Multi-Level/Mixed) Regression Models] package [50]. This checking procedure was applied to all the models presented below. Because of the high degree of collinearity between parasitaemia and gametocytaemia, these two variables were never fitted as response variables in the same model. The influence of parasitaemia or gametocytaemia on the day post-blood meal at which the maximum burden of oocysts or sporozoites was reached, as well as the day at which 10%, 50% or 90% of the blood-fed mosquitoes in a batch were detected as sporozoite carriers (i.e. EIP_10_, EIP_50_ and EIP_90_) was analysed using linear models. The average blood meal size of each mosquito batch was also added as an explanatory variable in the models. The influence of parasitaemia or gametocytaemia on mosquitoes’ blood meal size, oocyst burden and sporozoite density was analysed by fitting bird as a random factor into the models using lmer or glmer.nb (package: lme4 [51]) according to whether the errors were normally (haematin quantity, sporozoite density) or negative-binomially distributed (oocyst burden). Blood meal size and oocyst burden were added as explanatory variables in the models when the statistical unit was a mosquito and when the response variable might be influenced by these traits (see Table S2). The average blood meal size of the batch of mosquitoes was added as an explanatory variable in all models where the statistical unit was the batch of mosquitoes and when the response variable might be influenced by these traits (see Table S2). Maximal models, including all higher-order interactions, were simplified by sequentially eliminating non-significant terms and interactions to establish a minimal model [52]. The significance of the explanatory variables was established using a likelihood ratio test which is approximately distributed as a Chi-square distribution or an *F* test [53]. The significant Chi-square or *F* values given in the text are for the minimal model, whereas non-significant values correspond to those obtained before the deletion of the variable from the model. The different statistical models built to analyse the data are described in Table S2 in the electronic supplementary material.

## Results

### Parasitaemia and gametocytaemia of vertebrate host

**Fig. 1:**
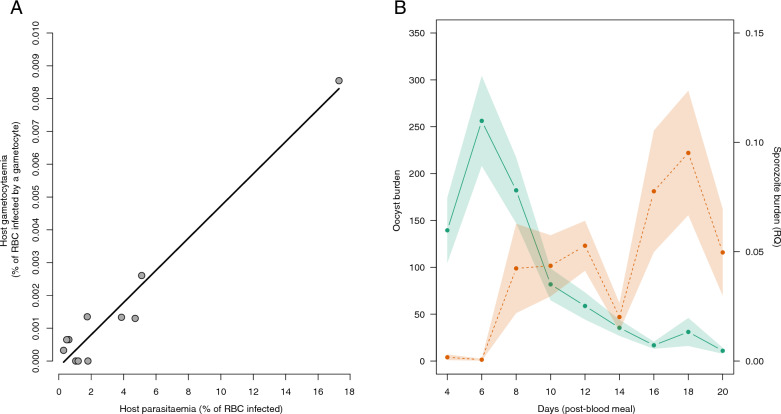
**A** Relationship between vertebrate host parasitaemia and gametocytaemia. Eleven individuals were included in this analysis. **B** Temporal dynamics of *Plasmodium* development in mosquitoes. Average oocyst burden (green, left axis) and sporozoite counts (salmon, right axis) at each dissection day. Green and salmon shadows represent standard error. The left axis represents the average number of oocysts counted per female. The right axis represents the amount of sporozoites quantified by qPCR

This experiment was carried out to investigate whether parasitaemia and gametocytaemia in the vertebrate host on the day of the infectious blood meal underpinned the parasite development within the invertebrate vectors. Eleven vertebrate hosts out of the 12 that were experimentally infected survived until day 12, allowing a range of parasitaemia and gametocytaemia values to be generated. The parasitaemia on the day of the mosquito blood meal (i.e. day 12 post-infection) followed an aggregated distribution (variance-to-mean ratio = 6.89, mean ± standard deviation [SD]: 3.47 ± 4.89). Three hosts had a parasitaemia value of less than 1%, four individuals had parasitaemia ranging between 1% and 2%, and four individuals had a parasitaemia value of over 3%, one of which presented a parasitaemia value of over 17% (Fig. 1A). The percentage of red blood cells infected with mature gametocytes was on average low (mean ± SD: 0.0015 ± 0.002) relative to what has been observed in other studies using the same *Plasmodium* strain. As reported in a previous study (e.g. [40, 54]), we observed a strong positive correlation between parasitaemia and gametocytaemia (model 1: *F* = 27.68, *P* < 0.001, adjusted *R*-squared: 0.724, Fig. 1A). As the conclusions of the subsequent analyses are the same whether parasitaemia or gametocytaemia was fitted as explanatory variable, we focus hereafter solely on the influence of parasitaemia. Parasitaemia measurement was indeed much less prone to slight error, since it only involved counting the number of infected red blood cells. In the case of gametocytaemia measurement, it was necessary to differentiate the parasite stages and their maturation levels, which at times can be somewhat subjective (at present, there are no molecular tools for identifying mature gametocytes). All analyses incorporating gametocytaemia instead of parasitaemia fitted as explanatory variables are presented in Appendix 1.

### Temporal dynamics of *Plasmodium* development in mosquitoes

The temporal dynamics of oocyst production inside the mosquito midgut were consistent between mosquito batches fed on the 11 infected birds (Fig. 1B, Fig. S1). Oocysts were detected in all batches of mosquitoes from the first day of dissection, i.e. the fourth day after the blood meal. The peak oocyst burden was reached between days 6 and 8 (Fig. 1B, Fig. S1). Subsequently, a swift reduction in the oocyst count was noted, such that by day 14 post-blood meal, only a residual burden ranging from 1% to 20% of the peak oocysts persisted. The first sporozoites appear on the head/thorax homogenate mainly after day 6 (Fig. 1B). However, for two mosquito batches, sporozoites were detected in some females on day 4 post-blood meal (Fig. S1). The peak concentration of sporozoites was reached between days 10 and 12 post-blood meal for half of the batches of mosquitoes and between days 16 and 18 for the other half (Fig. 1B, Fig. S1).

### Effect of vertebrate host parasitaemia on oocyst production

**Fig. 2:**
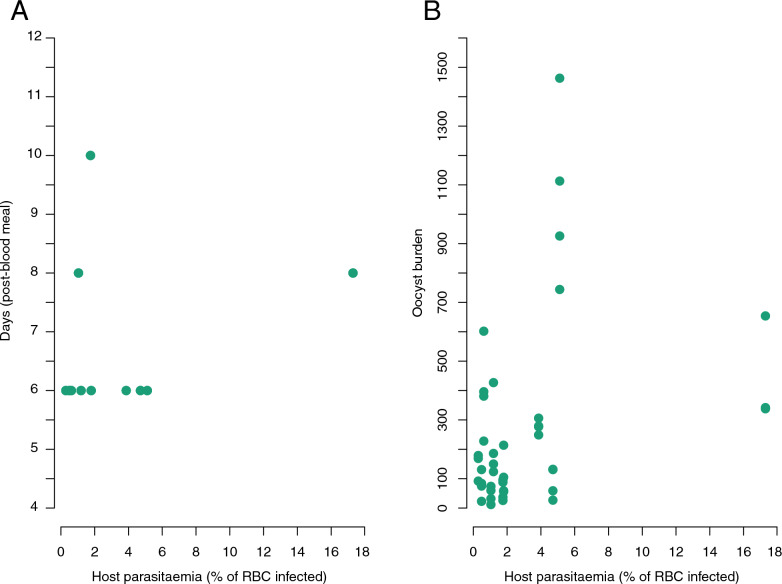
Relationship between vertebrate host parasitaemia and development of oocysts in the midgut of mosquitoes. **A** Parasitaemia did not significantly influence the number of days post-blood meal at which the maximum oocyst burden was reached. Each green dot corresponds to the batch of mosquitoes fed on a vertebrate host where the maximum oocyst burden was reached. **B** Parasitaemia of the bitten bird did not significantly influence the oocyst burden measured on the day when the oocyst burden was maximal in a focal mosquito batch. Each point represents the number of oocysts in the midgut of a dissected mosquito on the day when the mosquito batch associated with a bird, and therefore with a specific parasitaemia, reached maximum oocyst load

The size of the blood meal of mosquitoes was not influenced by the parasitaemia of the bird on which they fed (mean ± standard error [SE], 20.07 µg ± 0.055, model 2: *χ*^2^ = 0.256, *P* = 0.613). Since all mosquitoes already had oocysts in their midgut when we started the dissections (day 4 post-blood meal), we were unable to study the relationship between parasitaemia and the first appearance of oocysts. We showed that parasitaemia of vertebrate hosts had no influence on the day post-blood meal on which the oocyst peak was reached (model 3: *χ*^2^ = 0.456, *P* = 0.518, Fig. 2A) or on the density of oocysts reached during this peak (model 4: *χ*^2^ = 2.334, *P* = 0.127, Fig. 2B). While the blood meal size influenced the oocyst burden (the larger the blood meal, the greater the number of oocysts, model 4: *χ*^2^ = 12.65, *P* = 0.003), it did not influence the day post-blood meal on which the oocyst peak was reached (model 3: *χ*^2^ = 0.216, *P* = 0.655).

### Effect of vertebrate host parasitaemia on sporozoite production

**Fig. 3:**
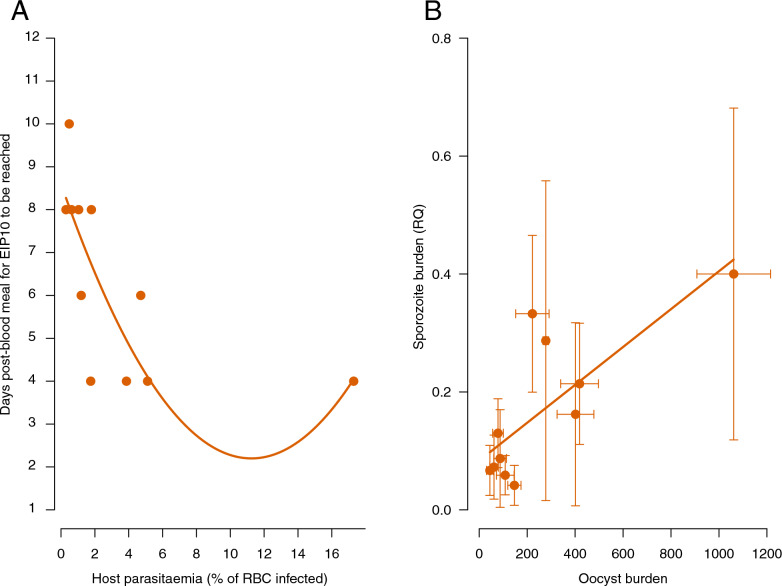
**A** Significant influence of parasitaemia on the day post-blood meal at which the EIP_10_ was reached (i.e. at least 10% of infected mosquitoes carried sporozoites). Each salmon dot corresponds to a batch of mosquitoes fed on a vertebrate host. **B** Positive relationship between average oocyst burden and average sporozoite load quantified in each batch of mosquitoes on the day of maximum oocyst and sporozoite load, respectively

The time required for sporozoites to be detected in the head/thorax homogenate of 10% of infected mosquitoes was influenced by the parasitaemia of the vertebrate hosts on which the mosquitoes fed (EIP_10_, model 5: *F* = 9.82, *P*= 0.016, Fig. 3A). Batches of mosquitoes that had bitten the most infected vertebrate hosts showed sporozoites as early as day 4 post-blood meal, which was up to 4–6 days earlier than females that had bitten the least infected vertebrate hosts (Fig. 3A). Fitting the quadratic term (parasitaemia^2^) slightly improved the model fit (model 5: *χ*^2^ = 6.45, *P* = 0.039), suggesting that the day on which the first sporozoites were detected was a decelerating polynomial function of vertebrate host parasitaemia. The average blood meal size of each mosquito batch did not influence EIP_10_ (model 5: *χ*^2^ = 2.18, *P* = 0.183). The effect of bird parasitaemia on the number of days after the blood meal required for 50% and 90% of the mosquitoes to become infectious was however no longer significant (EIP_50_, model 6: *χ*^2^ = 2.65, *P* = 0.148, EIP_90_, model 7: *χ*^2^ = 0.81, *P* = 0.397). The average blood meal size of each mosquito batch also had no effect (EIP_50_, model 6: *χ*^2^ = 1.93, *P* = 0. = 207, EIP_90_, model 7: *χ*^2^ = 0.39, *P* = 0.551). As previously reported, peak sporozoite concentrations in mosquitoes were reached between day 10 and 18 post-blood meal, but the time to reach the peak was not influenced by vertebrate host parasitaemia (model 8: *F* = 0.581, *P* = 0.468) or by blood meal size (model 8: *F* = 1.31, *P* = 0.727). The maximum sporozoite density reached during the peak was also not influenced by the parasitaemia of the vertebrate hosts (model 9: *χ*^2^ = 0.05, *P* = 0.815) or by the blood meal size (model 9: *χ*^2^ = 2.67 *P* = 0.102) but was influenced by the oocyst load reached during the oocyst peak (model 9: *χ*^2^ = 7.575, *P* = 0.006; Fig. 3B), where the higher the oocyst burden, the higher the sporozoite load.

## Discussion

The main finding presented in this study reveals a negative relationship between the intensity of malaria infection in the vertebrate host and the delay required after biting for the first mosquito vectors to become infectious. We showed that the time required for 10% of infected mosquitoes to become infectious (EIP_10_) was reached by the batches of vectors fed on the most infected vertebrate hosts as early as day 4 after the blood meal. However, taking a blood meal on highly infected vertebrate hosts did not affect the day after the blood meal required for 50% and 90% of mosquitoes to become infectious or the intensity of parasite infection within the mosquito. The number of oocysts and the concentration of sporozoites were not significantly affected by the vertebrate host parasitaemia or gametocytaemia (see Appendix 1 for gametocytaemia).

In several natural systems, such as *P. falciparum* and *An. coluzzii*, *P. falciparum* and *An. gambiae*, *Plasmodium vivax* and *Anopheles dirus*, or *Plasmodium mexicanum* and *Lutzomyia vexator,* it has been found that the oocyst burden in the invertebrate vector is positively correlated to the gametocyte density [32, 37, 39, 55]. This relationship, however, was fairly weak in many studies, and there was considerable variability in the number of oocysts associated with a given parasitaemia or gametocytaemia value (e.g. [55–58]). Density-dependent processes are at play in the regulation of the parasite journey within the vector, with major bottlenecks occurring during the life-stage transition from gametocyte to oocyst [7, 25, 59, 60]. Here, we observe that the maximum oocyst burden reached during peak oocyst development was not significantly influenced by parasitaemia or gametocytaemia (see Appendix 1 for gametocytaemia). The number of vertebrate hosts included in the study and the widely dispersed distribution of gametocytaemia (the majority of birds had very low gametocytaemia levels, variance-to-mean ratio = 38) could explain why the relationship between these two parameters was not statistically significant. Similar conclusions were however reported in a previous study carried out with the same biological system on 40 birds with a much wider range of gametocytaemia (i.e. 0.1–9% [40]). Therefore, our results and those published previously point in the same direction and strongly suggest that in the natural *P. relictum*/*Cx. pipiens* system, oocyst burden is not strongly influenced by gametocyte density [33, 40]. This could be explained by the fact that not all gametocytes counted in blood smears were yet infectious (i.e. ready to produce micro- or macro-gametes in the mosquito’s midgut). The mechanisms underlying gametocyte infectivity remain poorly understood. Although we know that gametocytes go through several stages of development before reaching the mature stage [61], we do not know whether the mature stage is systematically infectious.

 Parasitaemia and gametocytaemia in birds also had no influence on the maximum sporozoite density measured in the head/thorax of *Cx. pipiens.* However, we observed a positive relationship between maximum sporozoite density and the maximum average oocyst burden. Although the number of sporozoites produced per oocyst can vary from a few dozen to several thousand depending on the parasite/mosquito pair [62], a positive correlation between these two parasite stages is commonly reported [47, 55, 63–65]. The strength of the relationship between oocyst burden, sporozoite density and the rate of transmission of *Plasmodium* to vertebrate hosts is however difficult to assess due to the methodological complexity associated with the quantitative monitoring of the different parasite stages within a single mosquito. Nonetheless, a recent study conducted on both human and rodent malaria systems has clearly demonstrated a significant positive correlation between mosquito parasite load (oocyst and sporozoite stages) and the quantity of sporozoites injected during probing (i.e. inoculum size [65]).

In addition to inoculum size, the time lapse between mosquito infection (i.e. ingestion of gametocytes during the blood meal) and the moment when they become infectious (i.e. EIP) also has a major influence on the transmission dynamics of *Plasmodium*. Small changes in EIP can have a large effect on the number of mosquitoes living long enough to transmit parasites and subsequently on the number of hosts a vector might infect over its lifespan (e.g. [29, 66]). For the majority of *Plasmodium* species, EIP is on average between 8 and 16 days (see Table 1 in [67] and e.g. [68–71] for studies carried out on *P. relictum*). Several studies have shown that EIP can be highly heterogeneous between mosquitoes due to differences in nutrition (e.g. [66, 72–74]) and environmental conditions (e.g. temperature, see Fig. 1 in [42]); however, considerable variation remains even after accounting for these factors. Our results suggest that variation in EIP could be explained in part by the quantity of parasites ingested by the mosquitoes. Indeed, we detected the first sporozoites in batches of mosquitoes fed on the most infected hosts, on average 4–6 days earlier than in batches of mosquitoes fed on hosts characterized by lower parasitaemia and gametocytaemia. Interestingly, a previous study carried out on the same biological system, but with only three infected vertebrate hosts, highlighted a similar trend (see Fig. S1 in [75]). However, the effect of host parasitaemia on the evolution of the prevalence of infectious mosquitoes over time gradually diminished. We note that parasitaemia no longer had a significant effect on the EIP_50_ or EIP_90_. This result could be explained by the distribution of the quantity of parasites ingested by each mosquito within a batch of mosquitoes fed on the same host. Indeed, within a batch, the quantity of parasites ingested by each vector can vary greatly (e.g. Fig. 2 in [76]). The mosquitoes which ingested a greater quantity of parasites could correspond to those which we identified as being infectious as early as days 4–6 post-blood meal within the batches of mosquitoes fed on the most infected birds. To explore this question, a method would need to be developed to estimate the quantity of parasites ingested from the quantity of haematin excreted and the proportion of infected red blood cells in the host’s blood. To our knowledge, the only existing method of approximating the quantity of parasites ingested involves killing the mosquitoes before they digest the blood meal (e.g. [34]).

Several studies have suggested that the higher the parasite load in the mosquito midgut, the lower their survival rate (e.g. [23, 77]). In a situation of overcrowding, an adaptive strategy for the parasite to ensure transmission could therefore accelerate its development in order to colonize the mosquito’s salivary glands as quickly as possible, so that it can be transmitted to a new host before the vector dies. This pattern may be explained by density-dependent processes which may operate during the first steps of *Plasmodium* development within the mosquito midgut [9, 25, 59]. A high gametocyte density in the blood meal may lead to competition between parasites at different steps of early sporogony (e.g. fecundation, resources, colonization of the midgut wall). This competition could accelerate the development of all parasites and/or favour fast-growing parasite variants, both leading to early development of the first oocysts and, consequently, the rapid appearance of the first sporozoites. A theoretical study supports this hypothesis, but their results also suggest that above a certain density, the number of parasites in the mosquito midgut is so high that it could have a negative effect on the EIP (e.g. by negatively impacting oocyst survival rates or delaying oocyst burst, for example [27]).

## Conclusions

In summary, our study has demonstrated for the first time that the intensity of infection within the vertebrate host on which a mosquito feeds influences the EIP of *Plasmodium*. The first sporozoites were indeed observed in the batches of mosquitoes fed on the most infected hosts. It should be noted, however, that in this study we only used acutely infected hosts, and therefore with high parasitaemia. Indeed, as this was a pioneering study, we wanted to generate the widest possible range of infection intensities to detect a signal that might have been weak. In the field, the vast majority of vertebrate hosts are in the chronic stage of infection [78–80], and it would then be relevant to determine whether the results we obtained are translatable to milder parasitaemia. It should also be mentioned that in this study, to control as many parameters as possible (e.g. genetic background of the mosquitoes, health of the vertebrate hosts, parasite strains), we used a colony of mosquitoes maintained in the laboratory since 2017, domestic canaries from a professional breeder and a *Plasmodium* strain isolated from the field approximately 8 months prior to the experiment. It will now be relevant to compare our results with those that could be obtained using a more natural system. Finally, our study showed that oocysts may be detected much earlier than the literature would suggest (e.g. [25, 75]). A future study on the subject should begin monitoring earlier after the blood meal to determine the influence of the vertebrate host parasitaemia on the appearance of the first oocysts.

## Supplementary Information

Additional file 1: Fig. S1 Temporal dynamics of *Plasmodium* development in batches of mosquitoes for each bird bitten/parasitaemia. Average oocyst burden (green, left axis) and sporozoite counts (salmon, right axis) in mosquitoes for each bird (parasitaemia) at each dissection day. Green and salmon shadows represent standard error. The left axis represents the average number of oocysts counted per female. The right axis represents the amount of sporozoites quantified by qPCR.

Additional file 2: Table. S1 Blood meal rate per bird. BF = the number of fully blood-fed mosquitoes at the end of the experiment. NBF = the number of unfed mosquitoes at the end of the experiment. TOTAL = the number of uninfected mosquitoes placed in each cage at the beginning of the experiment. BM_RATE = the blood meal rate, which is the proportion of blood-fed mosquitoes for each bird.

Additional file 3: Table. S2 Description of statistical models used in the study. *N* = sample size. “Maximal Model” includes the complete set of explanatory variables. “Minimal model” is the model containing only the significant variables and their interactions. Avg_Haematin corresponds to the average blood meal size of each mosquito batch. Square brackets indicate variables fitted as random factors. Curly brackets indicate the error structure used (n: normal errors, b: binomial errors). The response variable was not transformed unless otherwise stated.

Additional file 4.

Additional file 5.

## Data Availability

All data supporting the conclusions of this paper will be available on the Dryad Digital Repository.

## Abbreviations

EIP: Extrinsic incubation period
RH: Relative humidity
