# Supplementary material for: Impact of the intensity of infection in birds on Plasmodium development within Culex pipiens mosquitoes
Source: Parasit Vectors. 2025 Feb 14;18:54. doi: 10.1186/s13071-024-06652-4 (PMC11827324; doi:10.1186/s13071-024-06652-4)
Supplement: Supplementary file 2 — Additional file 2: Table. S1 Blood meal rate per bird. BF = the number of fully blood-fed mosquitoes at the end of the experiment. NBF = the number of unfed mosquitoes at the end of the experiment. TOTAL = the number of uninfected mosquitoes placed in each cage at the beginning of the experiment. BM_RATE = the blood meal rate, which is the proportion of blood-fed mosquitoes for each bird. [file 13071_2024_6652_MOESM2_ESM.pdf]

Table S1

Bird	BF	NBF	TOTAL	BM_RATE
A	53	37	90	0,59
B	49	38	87	0,56
C	45	40	85	0,53
D	47	35	82	0,57
E	41	45	86	0,48
F	56	29	85	0,66
G	46	44	90	0,51
H	37	NA	90	NA
I	39	45	84	0,46
J	47	39	86	0,55
K	52	35	87	0,60
