# Supplementary material for: Impact of the intensity of infection in birds on Plasmodium development within Culex pipiens mosquitoes
Source: Parasit Vectors. 2025 Feb 14;18:54. doi: 10.1186/s13071-024-06652-4 (PMC11827324; doi:10.1186/s13071-024-06652-4)
Supplement: Supplementary file 3 — Additional file 3: Table. S2 Description of statistical models used in the study. N = sample size. “Maximal Model” includes the complete set of explanatory variables. “Minimal model” is the model containing only the significant variables and their interactions. Avg_Haematin corresponds to the average blood meal size of each mosquito batch. Square brackets indicate variables fitted as random factors. Curly brackets indicate the error structure used (n: normal errors, b: binomial errors). The response variable was not transformed unless otherwise stated. [file 13071_2024_6652_MOESM3_ESM.pdf]

Table S2

Variable of interest	Resp. variable	Model nb	N	Maximal model	Minimal model	R subroutine {error struct}
Gametocytaemia on the day of the mosquito blood meal	sqrt(Gam)	1	11	Parasitaemia	Parasitaemia	lm {n}
Blood meal size	Haematin	2	403	Parasitaemia + (1|Bird)	1 + (1|Bird)	lmer {n}
Day post-blood meal on which the oocyst peak was reached	Day	3	11	Parasitaemia	1	lm {n}
Oocyst burden	Oocyst	4	46	Parasitaemia + Haematin + (1|Bird)	Haematin + (1|Bird)	glmer.nb
Time required for sporozoites to be detected in 10% of infected mosquitoes	EIP10	5	11	Parasitaemia + Parasitaemia^2	Parasitaemia + Parasitaemia^2	lm {n}
Time required for sporozoites to be detected in 50% of infected mosquitoes	EIP50	6	11	Parasitaemia + Parasitaemia^2	1	lm {n}
Time required for sporozoites to be detected in 90% of infected mosquitoes	EIP90	7	11	Parasitaemia + Parasitaemia^2	1	lm {n}
Day post-blood meal on which the sporozoite peak was reached	Day	8	11	Parasitaemia	1	lm {n}
Sporozoite burden	Sporo	9	41	Parasitaemia + Haematin + Oocyst + (1|Bird)	Oocyst + (1|Bird)	lmer {n}
