# Supplementary material for: Impact of the intensity of infection in birds on Plasmodium development within Culex pipiens mosquitoes
Source: Parasit Vectors. 2025 Feb 14;18:54. doi: 10.1186/s13071-024-06652-4 (PMC11827324; doi:10.1186/s13071-024-06652-4)
Supplement: Supplementary file 4 — Additional file 4. [file 13071_2024_6652_MOESM4_ESM.docx]

**Additional file 2**

**Table. S1 Blood meal rate per bird**. “BF” gives the number of fully blood fed mosquitoes at the end of the experiment. “NBF” gives the number of unfed mosquitoes at the end of the experiment. “TOTAL” gives the number of uninfected mosquitoes placed in each cage at the beginning of the experiment. “BM_RATE” gives the blood meal rate which is the proportion of blood fed mosquitoes for each bird.

**Table. S2 Description of statistical models used in the study.** “N” gives the sample size. “Maximal Model” includes the complete set of explanatory variables. “Minimal model” gives the model containing only the significant variables and their interactions. Square brackets indicate variables fitted as random factors. Curly brackets indicate the error structure used (n: normal errors, b: binomial errors). The response variable was not transformed unless otherwise stated.

**Appendix 1: Influence of gametocytaemia instead of parasitaemia**

Effect of vertebrate host gametocytaemia on oocyst production

The size of the blood meal of mosquitoes was not influenced by the parasitaemia of the bird on which they fed (mean ± se, 20.07µg ± 0.055, X2=0.34, p = 0.55). Since all mosquitoes already had oocysts in their midgut when we started the dissections (day 4 post-blood meal), we were unable to study the relationship between gametocytaemia and the first appearance of oocysts. We showed that gametocytaemia of vertebrate hosts had no influence on the day post-blood meal on which the oocyst peak was reached (X2=0.96, p = 0.35) or on the density of oocysts reached during this peak (X2=2.70, p = 0.09).

Effect of vertebrate host gametocytaemia on sporozoite production

The time required for sporozoites to be detected in the head-thorax homogenate of 10% of infected mosquitoes was influenced by the gametocytaemia of the vertebrate hosts on which the mosquitoes fed (EIP10, F = 6.21, p = 0.037). Fitting the quadratic term (gametocytaemia²) slightly improved the model fit (X2 = 3.99, p = 0.05), suggesting the day at which sporozoites were detected was a decelerating polynomial function of vertebrate host gametocytaemia. Peak sporozoite concentration in mosquitoes were reached between day 10 and 18 post-blood meal, but the delay to reach it was not influenced by vertebrate host gametocytaemia (F = 0.96, p = 0.35). The maximum sporozoite density reached during the peak was not influenced by the gametocytaemia of the vertebrate hosts either (X² < 0.01, p = 0.93) but was influenced by the oocyst load reached during the oocyst peak (X² = 7.68, p = 0.005). The higher the oocyst burden, the higher the sporozoite load.
