# Supplementary material for: Impact of the intensity of infection in birds on Plasmodium development within Culex pipiens mosquitoes
Source: Parasit Vectors. 2025 Feb 14;18:54. doi: 10.1186/s13071-024-06652-4 (PMC11827324; doi:10.1186/s13071-024-06652-4)
Supplement: Supplementary file 5 — Additional file 5. [file 13071_2024_6652_MOESM5_ESM.docx]

**Additional file 1**

**Fig. S1** **Temporal dynamics of *Plasmodium* development in batches of mosquitoes for each bird bitten/parasitaemia**. Average oocyst burden (green, left axis) and sporozoite counts (salmon, right axis) in mosquitoes for each bird (parasitaemia) at each dissection day. Green and salmon shadows represent standard error. The left axis represents the average number of oocysts counted per female. The right axis represents the amount of sporozoites quantified by qPCR.
